# P-619. Relationship between Respiratory Syncytial Virus (RSV) Genetic Lineages and infant clinical phenotypes in Buenos Aires

**DOI:** 10.1093/ofid/ofaf695.832

**Published:** 2026-01-11

**Authors:** Dolores Acuna, Mercedes S Nabaes Jodar, Helena Brenes-Chacon, Luciana Montoto, Gretel Wenk, Vivian BOKSER, M Florencia Lucion, Maria del Valle Juarez, Maria Juliana Palau, Analia Vera, Cecilia Marta Vescina, Alejandra Alancay, Guadalupe Fernandez Gago, Natalia Trevino, Paula Eguiguren, Angela Gentile, M eugenia Ibanez, Liliana F Cannigia, Marta Costa, M Paula Della Latta, Matias Irazoqui, M Victoria Nadalich, Carla Ghihlione, Evelin Delarubia, Toro Rosana, Alicia S Mistchenko, Marie Wehenkel, Diego R Hijano, Octavio Ramilo, Asuncion Mejias, Mariana Viegas

**Affiliations:** Public Health Laboratory, Biology Department of the Faculty of Exact Sciences at Universidad Nacional de la Plata, La Plata, Buenos Aires, Argentina./ 2.National Scientific and Technical Research Council (CONICET), Argentina./3. Department of Infectious Diseases, St Jude Children’s Research Hospital, I Memphis, Tennessee, United States., La Plata, Buenos Aires, Argentina; 2.National Scientific and Technical Research Council (CONICET), Argentina., Buenos Aires, Buenos Aires, Argentina; St. Jude Children's Research Hospital, Germantown, TN; Molecular Biology Laboratory, Pedro de Elizalde Children´s Hospital, Ciudad de Buenos Aires, Argentina., Buenos Aires, Buenos Aires, Argentina; Molecular Biology Laboratory, Pedro de Elizalde Children´s Hospital, Ciudad de Buenos Aires, Argentina., Buenos Aires, Buenos Aires, Argentina; Hospital General Niños Pedro de Elizalde, buenos aires, Ciudad Autonoma de Buenos Aires, Argentina; Department of Epidemiology, Ricardo Gutiérrez Children's Hospital, Buenos Aires City, Argentina., Buenos Aires, Buenos Aires, Argentina; Ricardo Gutierrez Childrens Hospital, Buenos Aires, Ciudad Autonoma de Buenos Aires, Argentina; Microbiology Laboratory, Sor María Ludovica Children’s Hospital, La Plata, Buenos Aires, Argentina., La Plata, Buenos Aires, Argentina; Microbiology Laboratory, Sor María Ludovica Children’s Hospital, La Plata, Buenos Aires, Argentina., La Plata, Buenos Aires, Argentina; Microbiology Laboratory, Sor María Ludovica Children’s Hospital, La Plata, Buenos Aires, Argentina., La Plata, Buenos Aires, Argentina; Infectious Diseases Service, Sor María Ludovica Children’s Hospital, La Plata, Buenos Aires, Argentina., La Plata, Buenos Aires, Argentina; Infectious Diseases Service, Sor María Ludovica Children’s Hospital, La Plata, Buenos Aires, Argentina., La Plata, Buenos Aires, Argentina; Microbiology Laboratory, Sor María Ludovica Children’s Hospital, La Plata, Buenos Aires, Argentina., La Plata, Buenos Aires, Argentina; Infectious Diseases Service, Sor María Ludovica Children’s Hospital, La Plata, Buenos Aires, Argentina., La Plata, Buenos Aires, Argentina; Ricardo Gutierrez Children's Hospital, Buenos Aires, Ciudad Autonoma de Buenos Aires, Argentina; Alemán Hospital, Buenos Aires City, Argentina., Buenos Aires, Buenos Aires, Argentina; Alemán Hospital, Buenos Aires City, Argentina., Buenos Aires, Buenos Aires, Argentina; Alemán Hospital, Buenos Aires City, Argentina., Buenos Aires, Buenos Aires, Argentina; Alemán Hospital, Buenos Aires City, Argentina., Buenos Aires, Buenos Aires, Argentina; Instituto de Investigación de la Cadena Láctea (IDICAL) INTA-CONICET, Rafaela, Argentina, Rafaela, Santa Fe, Argentina; Interzonal General Acute Care Hospital San Roque, La Plata, Buenos Aires, Argentina., La Plata, Buenos Aires, Argentina; Interzonal General Acute Care Hospital San Roque, La Plata, Buenos Aires, Argentina., La Plata, Buenos Aires, Argentina; Interzonal General Acute Care Hospital San Roque, La Plata, Buenos Aires, Argentina., La Plata, Buenos Aires, Argentina; Interzonal General Acute Care Hospital San Roque, La Plata, Buenos Aires, Argentina., La Plata, Buenos Aires, Argentina; Virology Laboratory, Ricardo Gutierrez Children’s Hospital, Buenos Aires City, Argentina., Buenos Aires, Buenos Aires, Argentina; St. Jude Children's Research Hospital, Germantown, TN; St. Jude Children's Research Hospital, Germantown, TN; St. Jude Children's Research Hospital, Germantown, TN; St Jude Children's Research Hospital, Memphis, TN; 1. Public Health Laboratory, Biology Department of the Faculty of Exact Sciences at Universidad Nacional de la Plata, La Plata, Buenos Aires, Argentina. 2. National Scientific and Technical Research Council (CONICET), Argentina, La Plata, Buenos Aires, Argentina

## Abstract

**Background:**

RSV is the leading cause of lower respiratory tract infections (LRTI) in infants worldwide. Since the RSV Genotyping Consensus Consortium (RGCC) was established in 2023, studies have reported the circulation of different RSV lineages. Nonetheless, the clinical impact of RSV genetic variability on infants’ clinical presentation has not been well studied.

**Figure 1.**
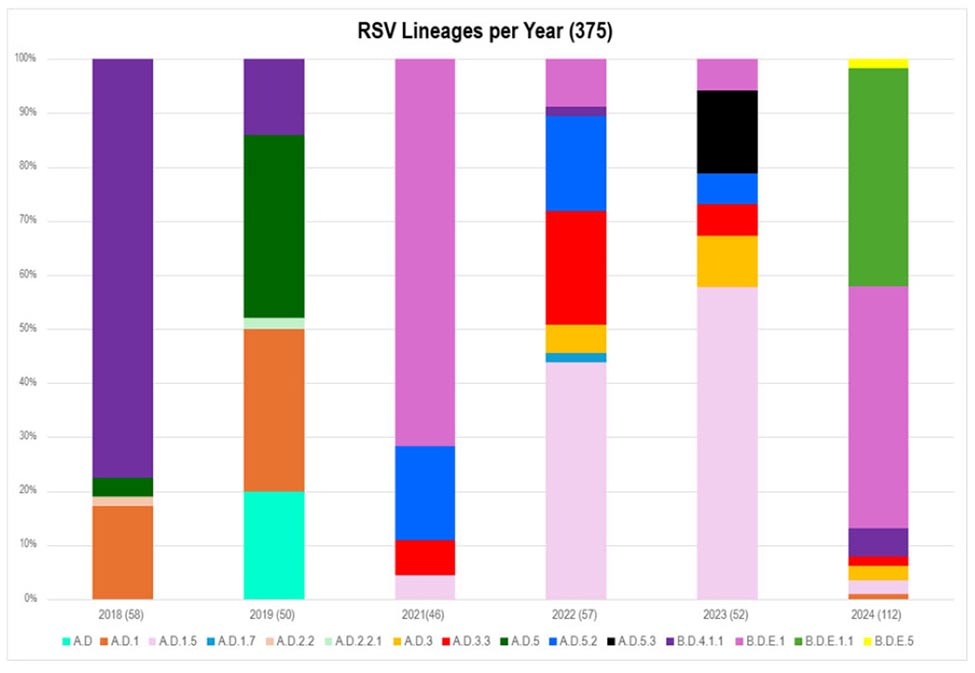
RSV Lineages per year. Percentage distribution of RSV lineages between 2018 and 2024 in Buenos Aires Metropolitan Area.

**Table 1.**
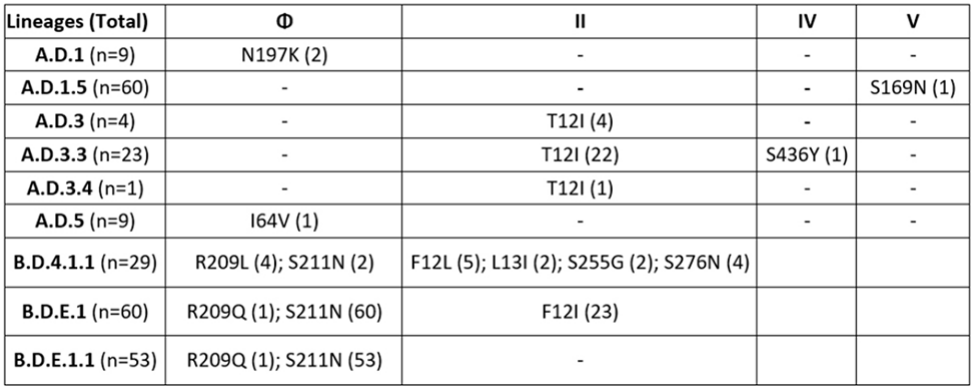
Amino acid substitutions in antigenic sites for RSV A and B lineages. The number of sequences with each substitution is indicated in parentheses. Reference sequences used: EPI_ISL_412866 (RSV-A) and EPI_ISL_1653999 (RSV-B).

**Methods:**

We analyzed 375 nasopharyngeal (NP) swab samples from infants with RSV hospitalized or evaluated at the outpatient clinics of 5 pediatric hospitals in Buenos Aires, Argentina, between 2018 and 2024. RSV whole genomes were sequenced by Illumina and Oxford Nanopore platforms. Genetic lineages were assigned using Maximum Likelihood trees following the RGCC reference alignments. The sequences were compared against RSV-A and RSV-B references to determine amino acid (aa) substitutions in the RSV F protein antigenic sites. Clinical data was collected and analyzed according to RSV genetic variability.

**Table 2.**
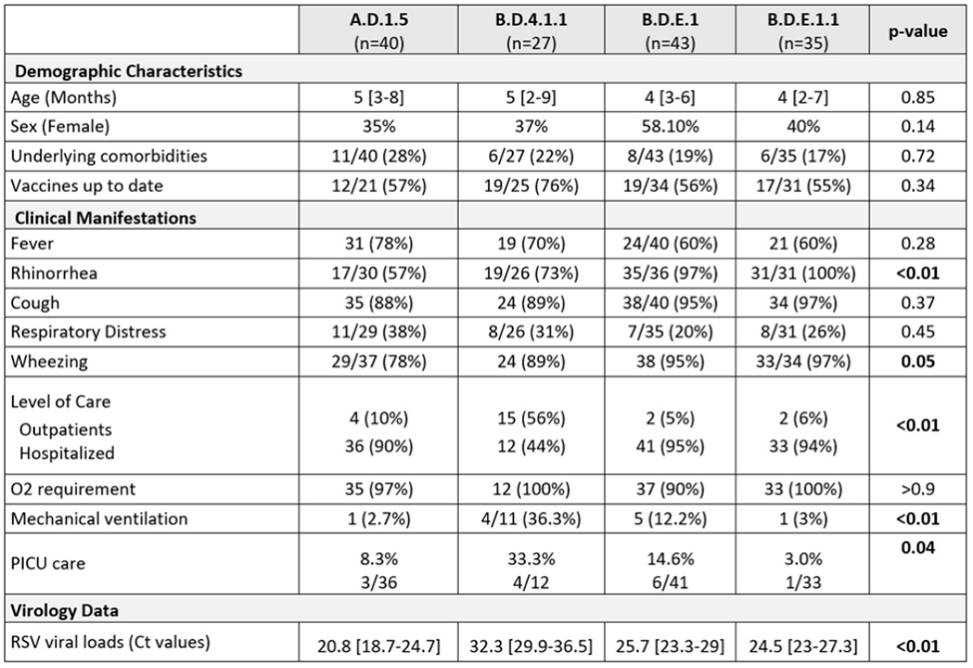
Comparison of demographic and clinical variables across the most frequent RSV genetic lineages. Categorical variables are presented as percentages or fractions and analyzed using Fisher’s exact test. Continuous variables are presented as median (25%–75% interquartile range) and were analyzed using the Kruskal-Wallis test with multiple comparisons. When data for a variable were not available for all patients, the total is expressed as a fraction based on the number of patients analyzed. PICU: Pediatric Intensive Care Unit. Ct RSV: Threshold Cycle in real time RT-PCR.

**Results:**

We identified a total of 15 genetic lineages, 11 for RSV-A and 4 for RSV-B. The predominant lineage before 2020 was B.D.4.1.1, while A.D.1.5, B.D.E.1, and B.D.E.1.1 became predominant after 2020 (Fig 1). Seven of the 15 lineages showed aa substitutions in antigenic sites φ and/or II, especially those pertaining to RSV B lineages (Table 1). There were no differences in age, sex or underlying comorbidities (i.e prematurity, chronic lung disease) according to the four most common genetic lineages (p >0.5). Infants infected with A.D.1.5 had rhinorrhea and wheezing less frequently and higher RSV loads as compared with the other three B sublineages (B.D.4.1.1, B.D.E.1, B.D.E.1.1) Table 2. On the other hand, B.D.4.1.1 infections were associated with greater need for PICU care and mechanical ventilatory support (p< 0.05).

**Conclusion:**

Multiple RSV lineages were detected over six different respiratory seasons in infants in Argentina. RSV B mutations were identified at a greater frequency than RSV A mutations including in site φ and site II. In addition, a specific RSV B sublineage was associated with greater disease severity. This data emphasizes the value of genomic RSV surveillance to monitor RSV activity and further our understanding on RSV pathogenesis and severity in children.

**Disclosures:**

Octavio Ramilo, MD, Merck: Advisor/Consultant|Merck: Grant/Research Support|Merck: Honoraria|Moderna: Advisor/Consultant|Pfizer: Advisor/Consultant|Pfizer: Honoraria|Sanofi: Advisor/Consultant Asuncion Mejias, MD, PhD, MsCS, Enanta: Advisor/Consultant|Merck: Grant/Research Support|Moderna: Advisor/Consultant|Pfizer: Advisor/Consultant|Sanofi-Pasteur: Advisor/Consultant

